# Validation of pathogen reduced plasmas from maxi‐pools combined with fast thawing

**DOI:** 10.1111/tme.13106

**Published:** 2024-11-05

**Authors:** Marja‐Kaisa Auvinen, Folke Knutson, Helena Löf

**Affiliations:** ^1^ Department of Clinical Immunology and Transfusion Medicine Uppsala University Hospital Blood Transfusion Center Uppsala Sweden

**Keywords:** fast thawing, maxi‐pools, pathogen reduced, plasma

## Abstract

**Objectives:**

Fast thawing for emergency situations and reduction of plasma wastage.

**Background:**

Evaluation of plasma units, pooled and pathogen reduced (PR) in “maxi‐pools” with amotosalen and UVA light, and fast thawing.

**Methods/Materials:**

Per replicate, 10 WB‐derived leukocyte depleted plasma units were frozen within 24 h at ≤ −25°C and stored for 7 days. After thawing, a maxi‐pool was constituted from the 10 units. After splitting into 4 sub‐pools of 650 mL, the sub‐pools were PR treated then split into 3 units resulting in 12 PR plasma units at 200 mL. Hundred and twenty PR plasma units were produced in total. The units were frozen at ≤ −25°C for 1 week, then thawed either in a fast plasma thawer for 5 min or in other control devices (17 to 23 min). FVIII:C, Fibrinogen, albumin, IgG, protein S and VWF were measured in plasma units, maxi‐pools and plasmas after PR treatment and thawing.

**Results:**

There was a statistically significant (*p* < 0.001) but still clinically acceptable (over the recommended levels of ≥0.5 IU/mL and ≥2 g/L) reduction of FVIII:C and Fibrinogen after PR with 69% and 87% recovery, respectively. Other proteins were not significantly affected by the processes.

**Conclusion:**

Pooling 10 plasma units before the PR treatment standardises volume and protein content of plasma units. Besides the economic value of generating 12 products for transfusion, this procedure combined with a thawing time of about 5 min is of value in emergency situations and may reduce plasma wastage.

## 1 INTRODUCTION

Many authors agree that early and timely plasma transfusion reduces mortality of trauma patients. High fresh frozen plasma to red blood cells (FFP:RBC) and platelets to red blood cells (PLT:RBC) ratios are associated with a survival benefit.^1^ Transfusion packs containing 4 units of erythrocytes, 4 units of plasma and 1 platelet unit have been adopted in our institution for trauma patient care. This practice had a detrimental effect on the outdating of plasma. In year 2005, 14% of plasmas for transfusion were outdated, and that figure raised to 25% in 2018. The plasma needed for the transfusion packs is thawed as soon as the order comes in, and in our institution the ward can return the unused units back to the blood bank. If there is no need for thawed plasma within 7 days after thawing, the plasmas are discarded. Also, if there is not enough documentation on how plasmas were stored at the ward department, or operating room, the plasmas are registered as outdated and discarded if not transfused. There is also a tendency in an acute situation to thaw extra plasma at the blood bank to be able to deliver the transfusion packs in a timely manner. These observations led to an interest in developing a “just in time” plasma supply methodology not involving previously thawed plasma. Devices using radio wave^2^ are becoming available and make it possible to rapidly thaw plasma in a dry environment without the additional preparation time of making a water bath available. This has also triggered a need to standardise the volume of plasma units to the lowest acceptable volume, that is, 200 mL, making the time thawing will take more predictable and shorter. This volume standardisation coupled with fast thawing allowed to decrease outdating of plasmas to 12%.

Clinicians also expressed a wish to have more standardised products with regards not only to volume but also content. In Sweden there is pooled solvent detergent (SD)‐treated plasma available (Octaplas, Stockholm, Sweden). However, it is registered as a medicine, and there is a separate legal framework for medicines and rules for procurement processes which are outside of blood banking. PR plasma made from pools smaller than 12 units is considered as a blood component,^3^ not a medicine. Pooled plasma is shown to be more homogeneous in contents of coagulation factors and other proteins^4^ as well as giving less transfusion reactions.^5^ The clinicians were looking for a product that could be used for patients reacting with allergic reactions against plasma. Also standardised volume for plasmapheresis was an advantage.

In our institution we have used a pathogen reduction (PR) technology, INTERCEPT® Blood System (Cerus Europe B.V., Amersfoort, The Netherlands) for platelets since 2007. The decision was taken after a fatal case of bacterial contamination. It was felt appropriate to introduce the same technology to a pooled product for plasma. We developed a 10‐unit plasma pooling technique allowing to optimise the use of pathogen inactivation (PI) processing sets and delivering 200 mL end products for transfusion.

The objective of our study was to assess, based on plasma quality parameters tested in‐vitro, a preparation procedure based on pools of 10 previously frozen plasma units subsequently split into volumes compatible with the process for PI treatment and thawed post‐frozen storage with a fast thawer (Conroy, Upplands Väsby, Sweden, CS201).

## 2 MATERIALS AND METHODS

### 2.1 Plasma preparation

**FIGURE 1:**
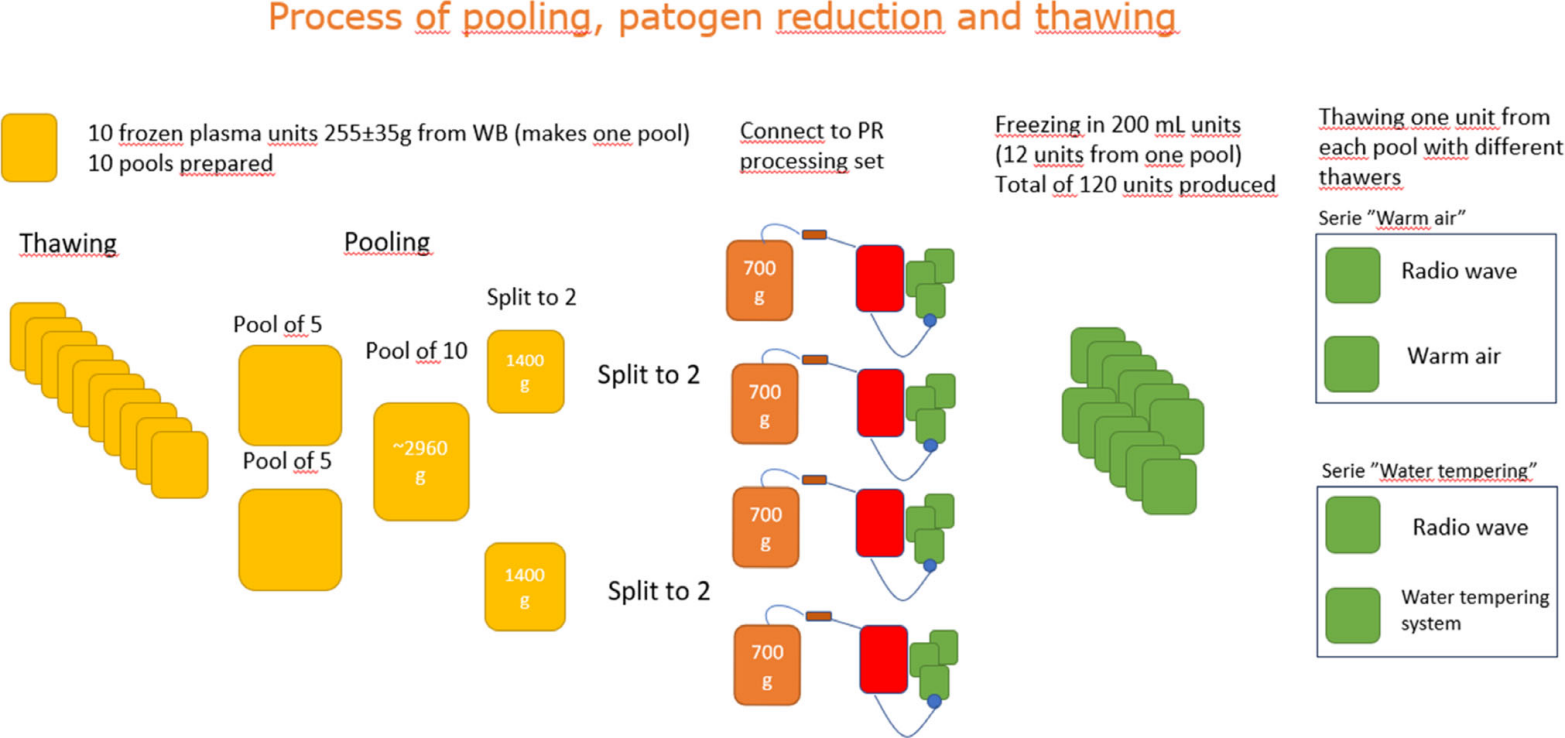
Flow chart for the preparation of PR plasma units. Where applicable, weights are indicated in grams (g). PR, pathogen reduced.

Whole blood units of 450 mL ± 10% were collected in blood packs (Macopharma, Tourcoing, France) containing CPD anticoagulant solution from 100 donors having given their consent for use of donated blood products for research. There was no Institutional Review Board approval required due to the study being related to a Quality Improvement project. Such projects do not require ethical approval because they are designed to improve processes and practices within existing standards of care. Plasma from study was not used for transfusion of patients. Blood donors have given their consent for such projects. The plasma was leukocyte‐depleted by filtration after separation from the red blood cells and the buffy‐coat using the filter from the blood pack system (In‐line Miniplas plasmafilter, Macopharma), quick frozen (Lundair, Helsingborg, Sweden) within 24 h and stored at ≤ −25°C and stored for 7 days. After thawing in a warm air operated device (Sahara Sarstedt, Nümbrecht, Germany), 10 groups of 10 A, B or AB units were weight‐selected to constitute maxi‐pools. The process is described in Figure 1. Maxi‐pools were obtained by sterile docking (TSCD, Terumo BCT, Tokyo, Japan) 2 × 5 plasma units to two double transfer packs (DONOpack, LMB, Schwaig, Germany) of 1,5 L capacity, themselves connected to a 3 L transfer pack (Macopharma). The total plasma volume obtained was above 2,6 L and was split after homogenization into 4 sub‐pools of approximately 650 mL retained in the 4 transfer packs of the 2 DONOpack units. Pooling was performed manually with careful mixing of the bags at each step, to obtain a homogeneous content in the pools before splitting.

### 2.2 Pathogen inactivation

Each sub‐pool of 650 mL plasma was sterile connected to an INTERCEPT plasma processing set for mixing with 15 mL of an amotosalen 6 mM solution and subsequent exposure to 3 J/cm^2^ of ultaviolet light (UVA) in an INT 100 Illuminator (Cerus, Amersfoort, The Netherlands). After reduction of the amotosalen concentration below 2 μM by adsorption to an in‐line compound adsorption device (CAD), 3 units of at least 200 mL PI‐treated plasma were obtained and quick frozen (Lundair) within 4 h from thawing. The 4‐sub pools of each maxi‐pool were treated in the same manner to obtain 12 PI‐treated fresh frozen plasma units (PI‐PFC).

### 2.3 Plasma thawing

The 12 PI‐PFC from each maxi‐pool were stored frozen at ≤ −25°C for 1 week and the units used for laboratory testing were either thawed in a CS201 (Conroy, Upplands Väsby, Sweden), one unit at a time, radio‐wave operated plasma thawer for approximately 5 min or in a water tempering system (Plasmatherm, Barkey Corporation, Woburn, MA, USA), 4 units at a time, or a warm air based device (Sahara, Sarstedt, Nümbrecht, Germany), 3 units at a time, for approximately 20 min to serve as control.

### 2.4 Plasma laboratory testing

Factor VIII (FVIII:C), Fibrinogen, von Willebrand Factor (VWF), protein S, albumin and IgG were measured in thawed plasma units, maxi‐pools and PI‐PFC units after PI treatment and thawing. Fibrinogen was also measured in the fresh plasma units before the initial freezing.

FVIII:C, Fibrinogen, VWF and protein S were assayed using chronometric methods on a STA‐R Max coagulometer (Diagnostica Stago, Asnières‐sur‐Seine France). Albumin and IgG were measured on a biochemical analyser cobas® pro, Roche. FVIII:C and Fibrinogen were selected because they are listed in the EDQM guidelines.^3^ FVIII: is also known as a labile factor, time and temperature dependent. VWF is an important contributor to primary haemostasis. protein S is an anticoagulant protein. Albumin and IgG are essential constituents of plasma. FVIII:C, Fibrinogen, protein S and VWF were shown to be affected to different degrees by PR processes.^6^, ^7^

### 2.5 Statistical analysis

If the individual plasmas were compared with the pools, a two‐sample Student's *t*‐test comparison of means with different variance was performed. In other situations, paired t‐test for comparison of the means was used.

## 3 RESULTS

### 3.1 Assessment of plasma quality

**FIGURE 2:**
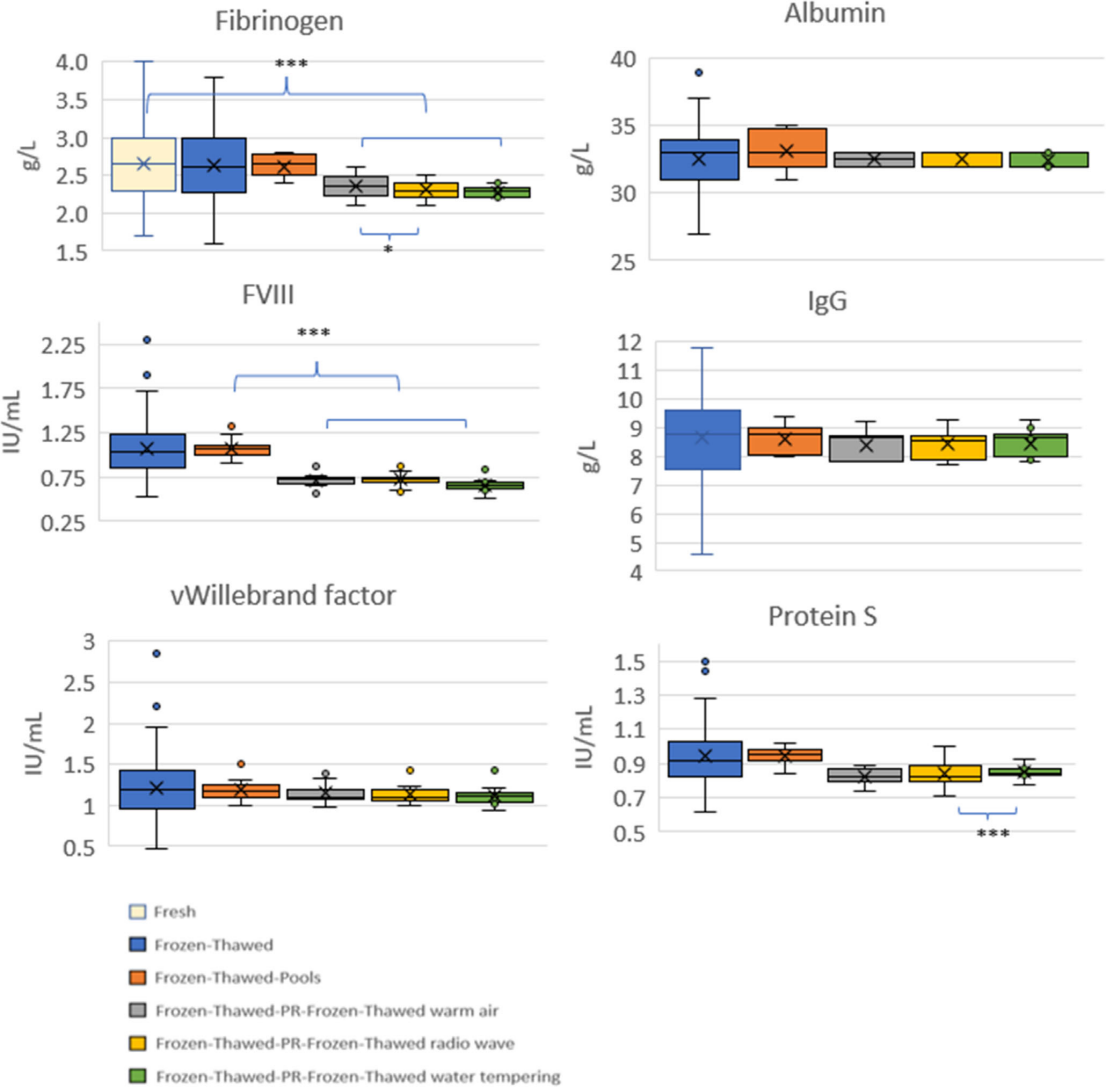
Boxplots of assays for Fibrinogen, FVIII, VWF, Albumin, IgG and protein S at the different phases of the process from the first freezing to thawing in different devices. When there are statistically significant differences between one and another phase of the process, it is shown with braces {where *p < 0.05, ***p < 0.001. The bracket [refers to all thawers as a group that is, the final thawing procedure. Minimum ꓕ, first quartile □, median____, mean X, third quartile □ and maximum values T.

The measurements of plasma quality parameters at the different stages of preparation are shown in Figure 2.

The requirements according to the Guide to the preparation, use and quality assurance of blood components (EDQM), 21st edition 2023^3^ for plasma, fresh frozen, PR are that it should contain for FVIII: on average, not less than 50 IU per 100 mL (i.e. 0.5 kIU/L) and for Fibrinogen ≥60% of the potency of the freshly collected plasma units.

FVIII:C content in frozen, thawed plasma was 1.1 kIU/L compared to 0.73 kIU/L in twice frozen, PR‐treated and thawed plasmas, which is 69% of the original activity (*p* < 0.05). Pools had a higher mean FVIII:C activity than the individual units, but this was not statistically significant. The fibrinogen content in frozen, thawed, PR‐treated‐frozen–thawed units was 2.2 g/L, which is 88% of the fresh units (2.7 g/L) (*p* < 0.05).

Comparison of quality parameters after thawing with different techniques is shown in Table 1. Fibrinogen was 2% higher when thawing in warm air compared to radio wave thawing, which is statistically but not clinically significant difference, since fibrinogen levels are still 87% of those with fresh blood. There were no significant differences in VWF, albumin or IgG concentrations when comparing the once frozen units with twice frozen and PR‐treated units. One marker, protein S was slightly lower on average when thawed with radio wave than with water tempering system (6%, *p* < 0.05). However, the testing method has variability in test results with CV 10%. The thawing tests were made on different dates, so the inter‐methodological variability in testing method may play role in observation.

**TABLE 1:** Results of the assays at the last thawing phase of the process in two series of thawing experiments.

Parameter	Plasma units from pools after A‐UVA PR, 7‐day ≤− 25°C storage and thawing—results per type of thawer (*N* = 10)			
	Radio wave in warm air series	Warm air	Radio wave in Water tempering series	Water tempering system
Factor VIII:C (kIU/L)	0.73 ± 0.08 [0.59;0.88]	0.72 ± 0.08 [0.56;0.88]	0.71 ± 0.05 [0.61;0.79]	0.66 ± 0.08 [0.52;0.83]
Fibrinogen (g/L)	2.31 ± 0.13 [2.1;2.5]	2.35 ± 0.16* [2.1;2.6]	2.32 ± 0.10 [2.2;2.4]	2.28 ± 0.08 [2.2;2.4]
VWF (IU/mL)	1.13 ± 0.13 [1.09;1.42]	1.15 ± 0.12 [0.9;1.38]	1.13 ± 0.08 [1.01;1.23]	1.11 ± 0.13 [0.94;1.43]
Albumin (g/L)	32.7 ± 0.5 [32.0;33.0]	32.6 ± 0.5 [32.0;33.0]	32.5 ± 0.5 [32.0;33.0]	32.4 ± 0.5 [32.0;33.0]
IgG (g/L)	8.41 ± 0.53 [7.70;9.20]	8.40 ± 0.52 [7.80;9.20]	8.42 ± 0.55 [7.70;9.30]	8.47 ± 0.51 [7.80;9.30]
Protein S (IU/mL)	0.88 ± 0.09 [0.71;1.00]	0.83 ± 0.05 [0.74;0.89]	0.81 ± 0.04 [0.74;0.88]	0.85 ± 0.04** [0.78;0.93]

*Note*: Mean ± SD [min;max].

Abbreviation: n.t, not tested.

* *p* < 0.05 when compared with radio wave.

** *p* < 0.01 when compared with radio wave.

As expected, pools were more homogeneous than individual plasmas, which is shown in the results by lower standard deviation and coefficient of variation with all the markers tested (Figure 2 **)**.

### 3.2 Assessment of plasma thawing

A time of around 30 min was observed for thawing frozen plasmas separated from whole blood when using a warm air thawer. After standardisation to 200 mL bags, after PRT, the thawing time decreased to about 20 min to 14–23°C. Water tempering system provides a thawing time of about 15 min for 200 mL standardised volume plasma units with a final temperature of 31–36°C after thawing. Radio wave technology thaws 200 mL plasmas within 5 min to 17–27°C.

## 4 DISCUSSION

The validation of PR plasmas from maxi‐pools combined with fast thawing showed that products meeting the European Guidelines and more standardised in volume and content can be obtained. Ang et al. have observed that the percentage decrease in FVIII:C is the highest among labile clotting factors, occurring naturally and independently of freezing and thawing procedures, with the greatest decrease within the first 24 h after plasma production.^8^ In our study there was a statistically significant but still clinically acceptable (over the recommended levels of ≥0.5 IU/mL kIU/L and ≥2 g/L) reduction of FVIII:C and fibrinogen after PR with 69% and 87% recovery, respectively. These are satisfactory results considering that the plasma has been frozen and thawed twice before the final thawing just before planned transfusion other proteins were not significantly affected by the processes. The reduction of FVIII:C concentration in PR plasma is acknowledged in the guidelines with a minimum level of 0.5 IU/mL instead of 0.7 IU/mL for untreated plasma. Such reduction has been observed in other studies^6^, ^9^ including comparisons with SD plasma.^7^ Studies showed that despite a diminished FVIII activity in PR plasma, the thrombin generation capacity was shown to be conserved equally well in plasma photochemically treated with amotosalen and UVA as in non‐treated plasma,^6^, ^7^, ^9^ demonstrating no significant effect of this technique on the global hemostatic properties of plasma requiring adequate functionality of FVIII:C and other factors. Clinical trials and hemovigilance programs suggest the observed loss of potency is of little clinical significance for the PR technique used.^10^

The main goal to assess the plasma freezing and thawing procedures was to decrease outdating of plasma. As previously mentioned, plasma outdates raised to 25% in 2018 mostly related to the return of the unused units back to the blood bank.

Just standardising the volume to 200 mL decreased outdating of plasmas to 12% in 2022, since it was more predictable how long‐time thawing would take. The use of a novel radio‐wave operated device allowed to consistently reduce the thawing time to 5 min. Reaching a final temperature of 17–27°C with radio wave is close to the temperature reached with warm air of 14–23°C our clinicians are used to. Warming to a higher temperature of 31–36°C with a water tempering technique is not perceived as a benefit. As a comment, after this study we have also changed our freezing process. Plasma bags are frozen on flat bed instead of vertical freezing leading to more uniform frozen units, which reach end temperature faster.

The second driver for changing the plasma procedure was to address the clinicians wish to have more standardised products with regards to content and volume. Pooled plasma is shown to be more homogeneous in contents of coagulation factors and other proteins as well as giving less transfusion reactions. The standardisation of volume and plasma protein content was observed in other studies in which mini‐pools of 5 plasmas were constituted before proceeding with the PR treatment using amotosalen and UVA light.^9^, ^11^ The pooling effect allows as well to limit the number of units not meeting guidelines as seen in these studies and our protocol which was using a large pool size of 10 units before treatment. One‐year quality control data showed no values below the EDQM required thresholds.

The plasma content, such as coagulation factors or other proteins, has been used as a surrogate marker for plasma quality. Frozen plasma can vary between units depending on the source of plasma as well as separation, freezing and thawing processes, so studies usually compare the quality of plasma at different stages with freshly donated plasma. Due to the in‐vitro nature of the study, there are limits with extrapolation of the use of maxi‐pool derived PR plasmas in clinical practice. The standardised volume and content can be considered as an aid to the clinicians for adequate dosing, but the potential benefits to the patients were not evaluated through a randomised clinical trial. Hemovigilance data are recorded with a passive system in our institution. Fast thawing directly impacted the level of discards but the time to availability of transfusion packs was not measured. We did not conduct a cost‐effectiveness evaluation of the change of practice.

In our institution we have used PR technology for platelets since 2007. The decision was taken after a fatal case of bacterial contamination. It was natural to introduce the same technology to a pooled product for plasma.

## 5 CONCLUSION

Pooling of plasmas results in standardised volume and content of plasma for transfusion compared with the individual plasmas. Freezing the initial plasma shortly after component separation keeps the quality of plasma on the same level as it would be for fresh plasma. Freezing allows to plan the further steps of the process according to the needs for products and access to staff performing the manual pooling procedures. The pooling phase of the process and the second freezing step maintain the quality of plasma, especially when throughput time is planned to be short. The introduction of PR technology for pooled plasma was straight forward since the equipment and method were already in use for platelets.

Fibrinogen and FVIII levels were acceptably maintained in the final products and fulfilled the requirements with good margins. Other proteins such as IgG, albumin or VWF were well maintained and the concentrations were not significantly different from the ones in once frozen units.

The standard volume of 200 mL allows together with new freezer techniques and future thawing devices to optimise the procedure and achieve optimal plasma supply for acute situations in a timely manner.

## AUTHOR CONTRIBUTIONS

MKA has contributed to study design, data collection, analysis and interpretation of result, and manuscript preparation. FK has contributed to study conception and design. HL has contributed to data collection and analysis, and manuscript preparation.

## FUNDING INFORMATION

Funding to partially cover the cost of laboratory assays for this study was received from Cerus Europe B.V.

## CONFLICT OF INTEREST STATEMENT

The authors have no competing interests.
